# Supplementary material for: Impact of V-box insertion on promoter activity and virus-inducibility in transgenic Arabidopsis thaliana
Source: PeerJ. 2025 Oct 31;13:e20178. doi: 10.7717/peerj.20178 (PMC12581914; doi:10.7717/peerj.20178)
Supplement: Supplemental Information 1 — Std. Dev.: Standard deviation; b=C.V. %: Coefficient of variation percentage; PRESS: Predicted residual error sum of squares; Adj R-Squared: Adjusted R-squared; Pred R-squared: Predicted R-squared; Adeq Precision: Adequate precision. [file peerj-13-20178-s001.docx]

**Table S1** The Model fit goodness and prediction

| Std. Dev. | 0.042 | R-Squared | 0.995 |
| --- | --- | --- | --- |
| Mean | -0.369 | Adj R-Squared | 0.993 |
| C.V. % | 11.441 | Pred R-Squared | 0.989 |
| PRESS | 0.321 | Adeq Precision | 85.873 |
