# Supplementary figures and images for: Impact of V-box insertion on promoter activity and virus-inducibility in transgenic Arabidopsis thaliana
Source: PeerJ. 2025 Oct 31;13:e20178. doi: 10.7717/peerj.20178 (PMC12581914; doi:10.7717/peerj.20178)

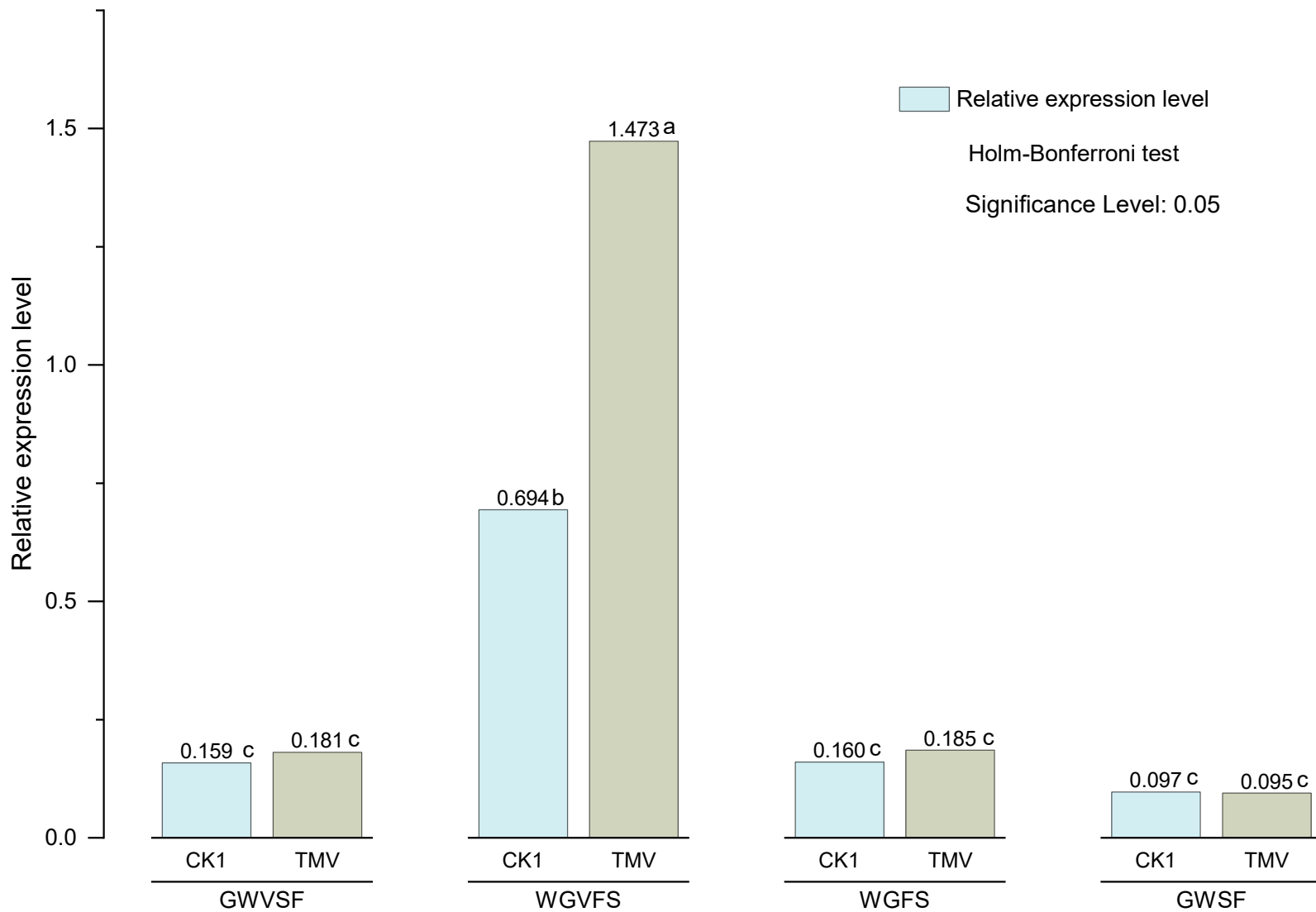

Supplement: Supplemental Information 4 — WGVFS: a modified promoter with the V-box inserted at the WGFS promoter midstream position; GWVSF: another modified promoter with the V-box inserted at the WGFS promoter midstream position. CK1 indicates the promoter transgenic plants not treated without inducer treatment. Transcriptional expression levels are relative values, calculated as the ratio of the expression level of each promoter to that of the CaMV35S promoter. Numbers on the bars in the figure indicate the averages of transcriptional expression levels, and different letters after the numbers indicate significant differences at the 0.05 level. [file peerj-13-20178-s004.pdf]

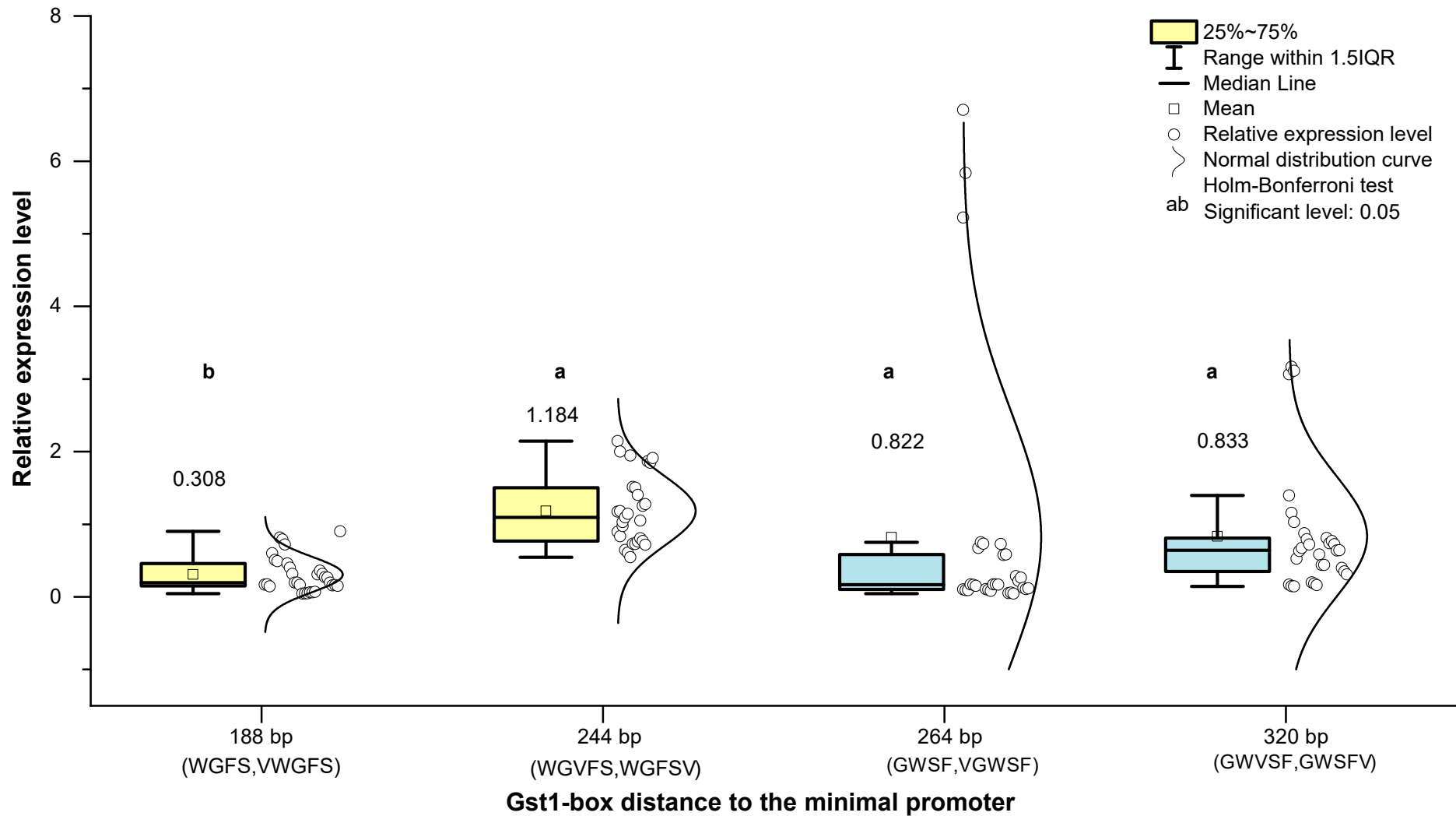

Supplement: Supplemental Information 5 — Each point corresponds to the average of the relative expression levels of a specific promoter and V-box position (including duplicates). [file peerj-13-20178-s005.pdf]

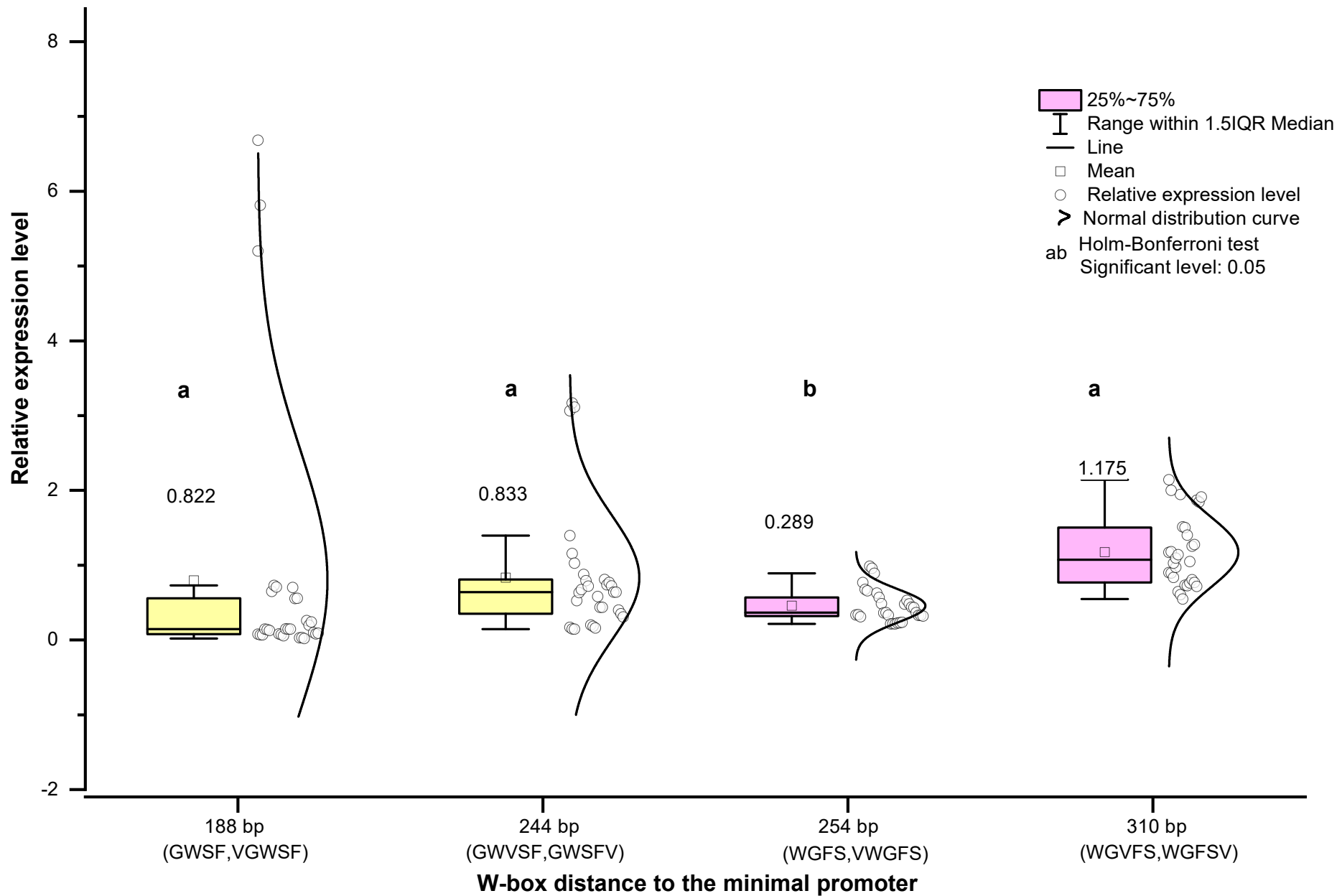

Supplement: Supplemental Information 6 — Each point corresponds to the average of relative expression levels for a specific promoter and V-box position including replicates. [file peerj-13-20178-s006.pdf]

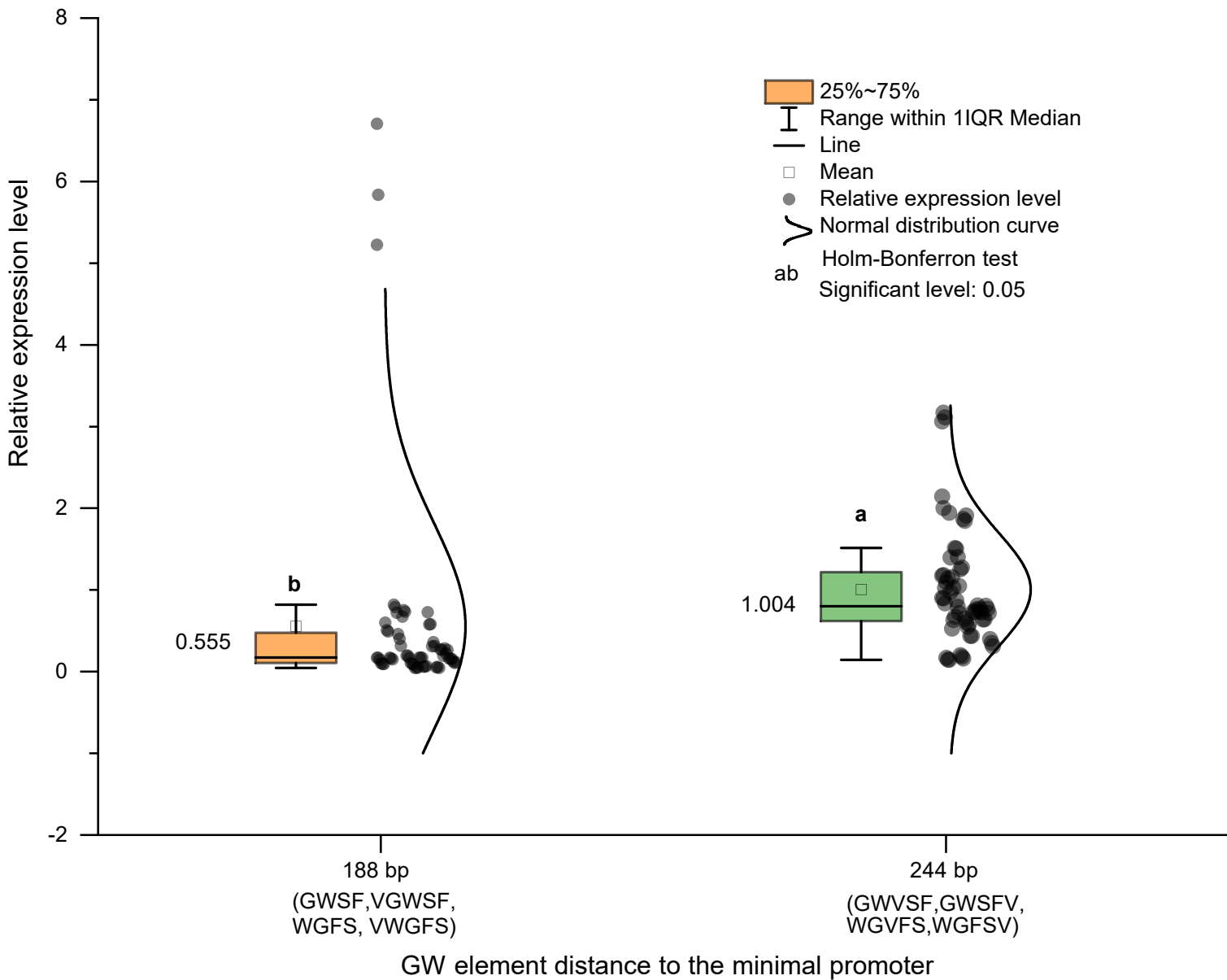

Supplement: Supplemental Information 7 — Each point corresponds to the average of relative expression levels for a specific promoter and V-box position including replicates. [file peerj-13-20178-s007.pdf]

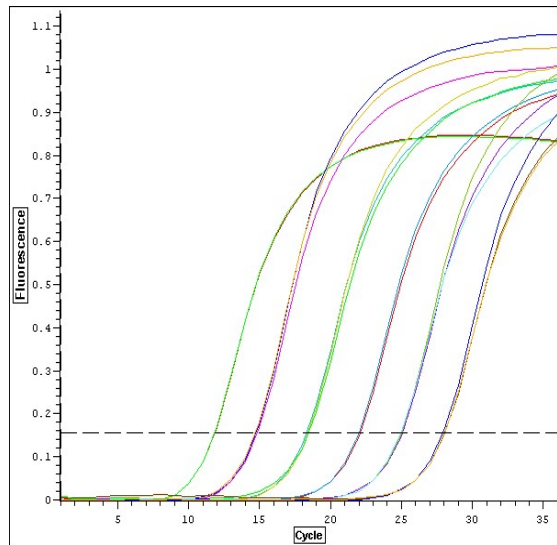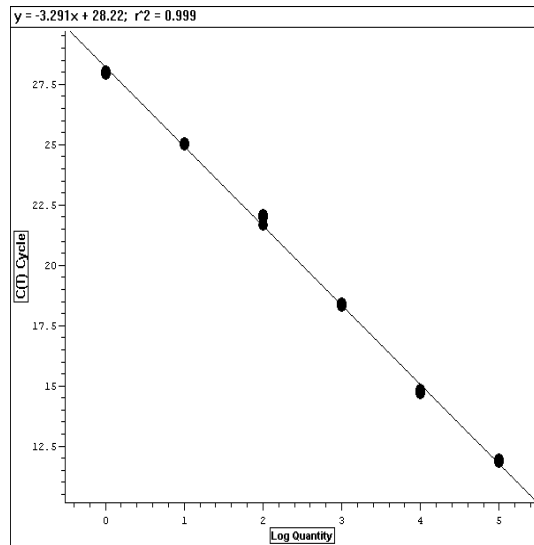

Fig. S4 The amplification efficiency of *gus* gene and standard curve

Supplement: Supplemental Information 8 [file peerj-13-20178-s008.pdf]

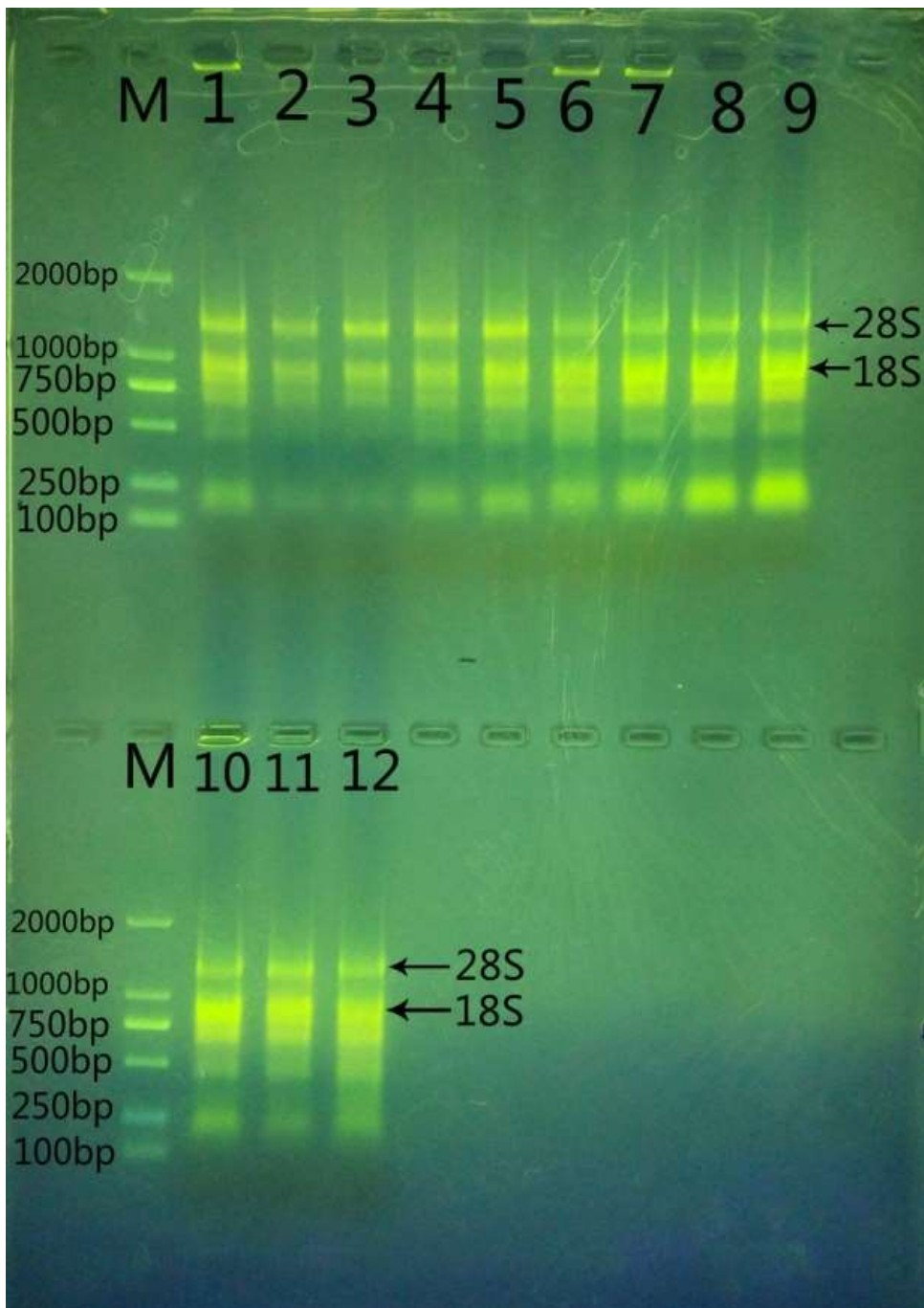

Fig.S5 Total RNA detection in *Arabidopsis thaliana*

M lane: DL 2000 DNA Marker

Supplement: Supplemental Information 9 — M lane: DL 2000 DNA Marker. [file peerj-13-20178-s009.pdf]
